# Epilepsy in spinocerebellar ataxia type 8: a case report

**DOI:** 10.1186/s13256-019-2270-x

**Published:** 2019-11-15

**Authors:** Arun Swaminathan

**Affiliations:** Department of Neurological Sciences Comprehensive Epilepsy Program, University of Nebraska Medical Center, South 42nd Street and Emile Street, Omaha, NE 68198 USA

**Keywords:** Epilepsy, SCA8, Neurodegenerative disease, Status epilepticus

## Abstract

**Background:**

Spinocerebellar ataxia type 8 is an uncommon genetic condition and presents with gait disturbances, ataxia, dysarthria, nystagmus, and cognitive and psychiatric abnormalities. Seizures are extremely uncommon in the spinocerebellar ataxias and have been reported only once before in a patient with spinocerebellar ataxia type 8. This case report highlights the need to evaluate spells in patients with a known neurodegenerative or genetic disease to exclude seizures, and it stresses the importance of timely diagnosis and therapy.

**Case presentation:**

The patient was a 22-year-old Caucasian woman with known spinocerebellar ataxia 8 since age 10 years. She was admitted to our hospital with new-onset left hemiparesis and encephalopathy in addition to chronic occurrence of multiple spells of confusion and oromanual automatisms with postictal lethargy. Testing confirmed that she was having recurrent seizures with episodes of nonconvulsive status epilepticus. Urgent treatment with antiepileptic therapy was initiated; her seizures resolved shortly thereafter, and her mental status improved. Her left hemiparesis has improved; she remains seizure-free; and she has returned to her baseline antiepileptic medications following physical therapy.

**Conclusions:**

Seizures have been reported extremely rarely in association with spinocerebellar ataxia 8, but they must be considered in the differential diagnosis of patients with spells of altered awareness, especially in those with a known neurodegenerative or genetic condition. Clinicoradiological correlation with symptoms can help expedite diagnosis and treatment. Expert consultation with epileptologists at the earliest signs can help establish the diagnosis quickly, minimize morbidity, and enhance recovery.

## Background

Spinocerebellar ataxia (SCA) is a relatively uncommon condition with a prevalence estimated to be about 2.7 per 1 million population [1]. Clinical features typically include ataxic gait, nystagmus, and cerebellar symptoms such as incoordination. SCA type 8 (SCA8) is among the less commonly seen types of ataxia but is being diagnosed increasingly more often due to increased genetic testing and raised awareness among clinicians. Ataxia, tremor, gait difficulties, nystagmus, and myoclonus are among the commonly seen symptoms in SCA8, but variable manifestations involving cognitive and psychiatric symptoms have been reported, making diagnosis challenging [2, 3]. The patient in this report was diagnosed with SCA8 and had chronic, recurring spells that were confirmed to be epileptic seizures after she was found to have nonconvulsive status epilepticus. Only a single previous case of myoclonic epilepsy was reported in a patient with SCA8 [4]. To the author’s knowledge, the present report describes the first confirmed case of SCA8 with focal onset epilepsy.

## Case presentation

A 22-year-old right-handed Caucasian woman with a known diagnosis of SCA8 since the age of 10 years was admitted to our university medical center with encephalopathy and left-sided hemiparesis of unclear cause over the last 3 months. She had been diagnosed with SCA8 at 10 years of age after presenting with ataxia and gait difficulties that progressed rapidly. She was diagnosed at the Children’s Hospital of Nebraska after genetic testing confirmed the diagnosis. She continued to see the geneticists there for management of her condition. Her family was also tested and was found to be negative for genetic mutations, confirming the patient as the only affected family member, probably from a sporadic mutation. Neuropsychological testing was not performed at the time, but the patient’s family reported that she had an average IQ and was able to speak normally and perform daily functions without difficulty.

**Fig. 1:**
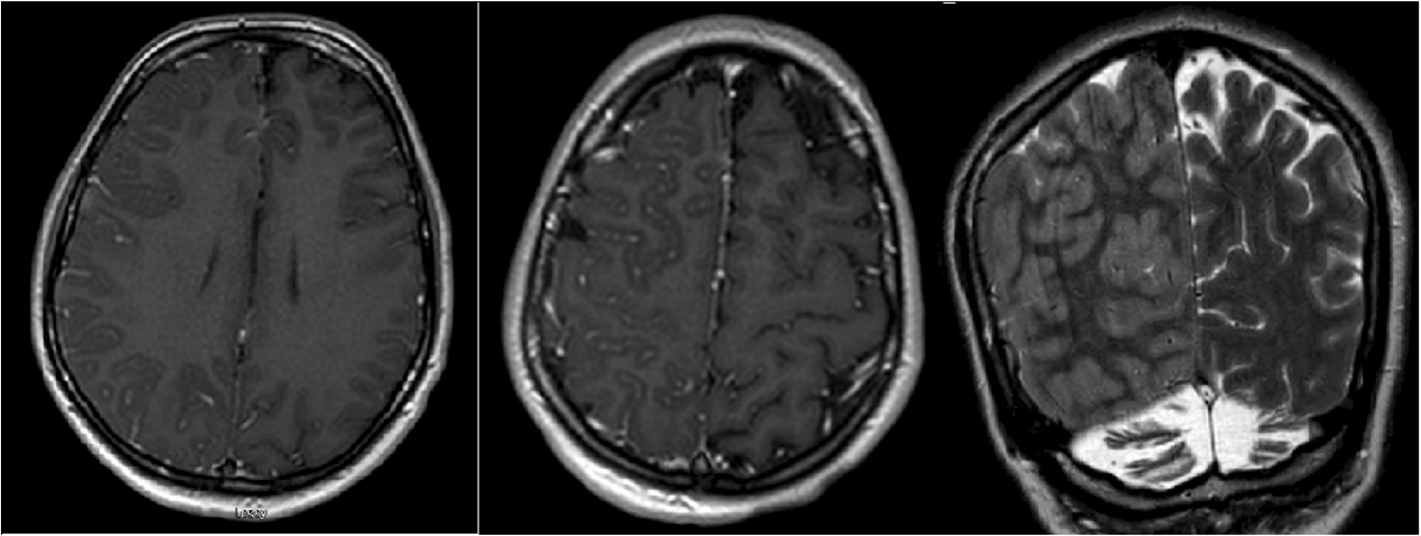
Magnetic resonance imaging scans showing right hemispheric leptomeningeal enhancement and edema in all three panels: T1-weighted sequence images with contrast in left and central panels and T2-weighted sequence image on right panel

**Fig. 2:**
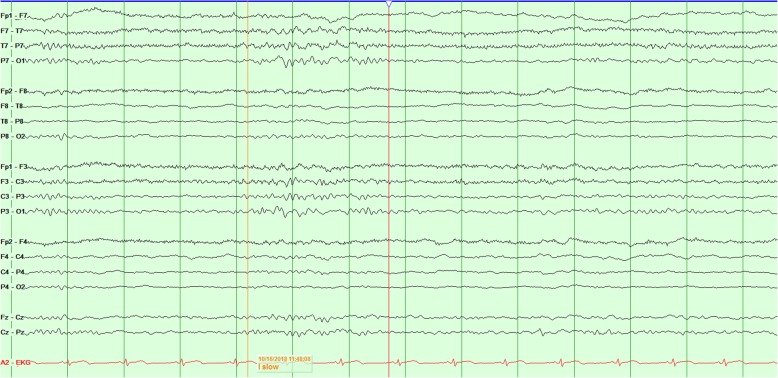
Interictal electroencephalography with a bipolar montage showing diffuse slowing with greater right hemispheric slowing and attenuation

**Fig. 3:**
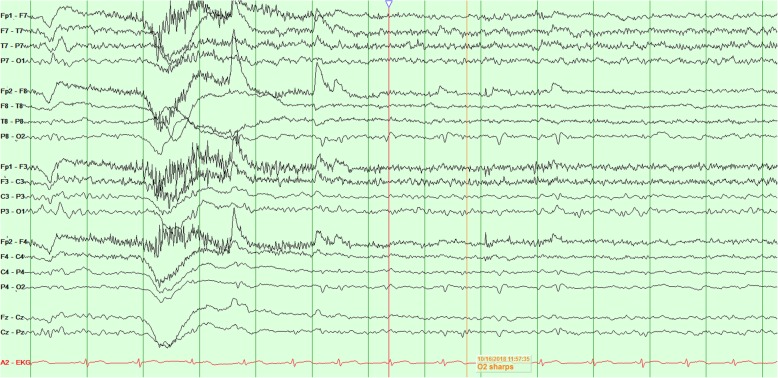
Interictal electroencephalography with a bipolar montage showing quasiperiodic epileptiform discharges at O_2_ electrode

The patient’s physical examination on admission at our center revealed encephalopathy with left hemiparesis without obvious visual field deficits or other cranial nerve deficits. Magnetic resonance imaging scans revealed leptomeningeal contrast enhancement and edema over the right hemisphere (Fig. 1). The results of lumbar puncture and resulting cerebrospinal fluid studies were unremarkable. A routine electroencephalogram (EEG) revealed independent slowing of both hemispheres, with the right hemisphere showing greater focal slowing and attenuation as well (Fig. 2). Right posterior quadrant epileptiform discharges from an O_2_ electrode were occasionally seen in a quasiperiodic manner (Fig. 3). Given these findings, the patient was started on levetiracetam therapy to treat potential epileptogenicity from the right posterior quadrant.

**Fig. 4:**
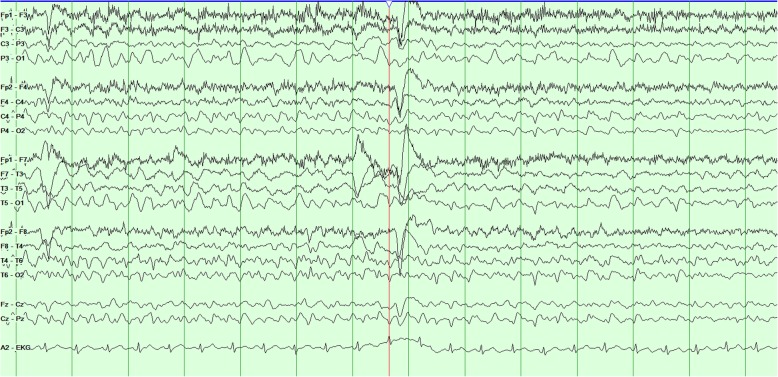
Ictal electroencephalography with bipolar montage showing seizure activity with involvement of bilateral posterior quadrant electrodes with rhythmic high-amplitude delta activity consistent with nonconvulsive status epilepticus

**Fig. 5:**
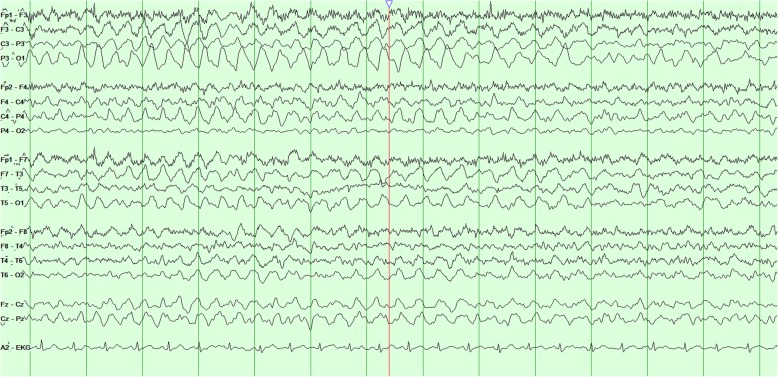
Ictal electroencephalography with bipolar montage showing seizure activity with involvement of bilateral posterior quadrant electrodes with rhythmic high-amplitude delta activity consistent with nonconvulsive status epilepticus

**Fig. 6:**
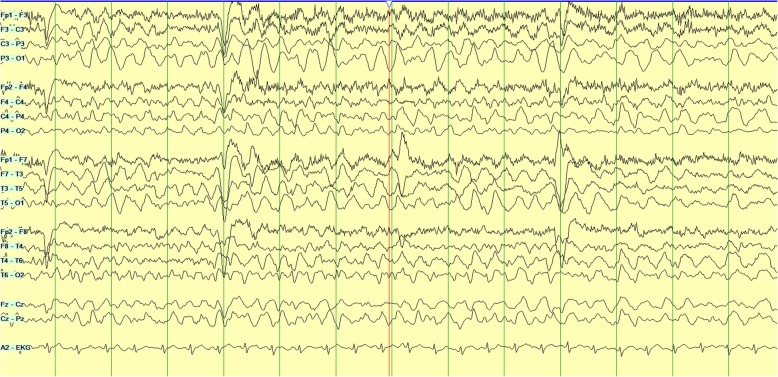
Ictal electroencephalography with bipolar montage showing seizure activity with involvement of bilateral posterior quadrant electrodes with rhythmic high-amplitude delta activity consistent with nonconvulsive status epilepticus

Two days later, she was noted to have frequent spells of confusion and decreased awareness. Owing to concern for ongoing seizures, she was connected to a long-term video EEG monitor for diagnosis. Video recordings captured multiple spells, each lasting 2–3 minutes, of loss of awareness with left gaze deviation and oromanual automatisms and staring with postictal lethargy and confusion consistent with clinical seizures. EEG captured posterior quadrant onset from both left and right hemispheres consistent with electroclinical seizures. A clear lateralization of onset was not seen with many of these seizures, owing to rapid bilateral involvement of both posterior quadrants (Figs. 4, 5, and 6). Many of these seizures occurred frequently over a 2–3-hour period, meeting criteria for status epilepticus. She was started on lacosamide therapy, and her dose of levetiracetam was increased. Her seizures resolved within a few hours of increasing her antiepileptic therapy.

Her family revealed that she had been having similar spells since the age of 12 years and that they had witnessed at least 15–20 similar spells in the past that were not previously recognized as seizures. Many of these spells were associated with nausea and vomiting, findings that were not captured on our video EEG recordings.

She continued to show improvement in mental status, and her left hemiparesis showed progressive improvement over the next few days with antiepileptic therapy and physical therapy. She was discharged to a rehabilitation facility on lacosamide and levetiracetam, continues to return for follow-up as an outpatient, and is currently doing well without any further seizures. When she was seen 3 months after her admission, her left hemiparesis had improved, and she had returned to her previous mental baseline.

## Discussion and conclusions

SCA8 is a neurodegenerative disease inherited in an autosomal dominant pattern and should be considered in the differential diagnosis of progressive ataxias. SCA8 is distinct in several aspects from other genetic progressive ataxias [5]. Most notably, the symptomatology of SCA8 is remarkably variable and at times episodic [3, 6].

Gait difficulty, ataxia, dysarthria, dysphagia, sensorimotor manifestations, nystagmus, and cognitive difficulties are reported in many patients with this condition [3, 7]. There is a single prior case report of epilepsy in a patient with SCA8, but that patient was reported to have myoclonic epilepsy, in contrast to our patient, who presented with focal epilepsy of posterior quadrant onset [4]. Seizure-like episodes in SCA8 have also been reported before, but these have been EEG-negative and suggestive of dystonic episodes or other movement disorders, such as chorea, rather than true epileptic seizures [8, 9].

Seizures are a known feature of SCA10 and SCA13 but have been reported only once before in SCA8 [10, 11]. The only other case of epilepsy in SCA8 was of the myoclonic epilepsy subtype. Our patient has focal epilepsy of posterior quadrant onset, which makes her presentation a unique phenotype of SCA8. We hypothesize that her SCA8 is probably causative of her epilepsy rather than a coincidental finding, because she has an unusual semiology for her seizures in addition to other features of SCA8, which suggest association by causation rather than coincidence. Moreover, it is highly unlikely that she would have an uncommon occipital seizure onset as a coincidental finding with her rare genetic condition, rather than having the occipital epilepsy as a part of its phenotype. Pure occipital onset epilepsy is relatively uncommon, and for it to occur concurrently in a patient with a rare genetic syndrome suggests genetic causation of this occipital epilepsy rather than coincidence. The patient has no family history of epilepsy, and no other family members have SCA8. Our patient does have presenting ataxia, which resulted in her initial diagnosis based on genetic testing. She also had multiple witnessed spells during her lifetime since the age of 12 years, which were retrospectively recognized as seizures. Her semiology of staring, loss of awareness, and automatisms with nausea and vomiting is consistent with posterior quadrant and occipital involvement in her seizures. The author hypothesizes that she may have had multiple unrecognized seizures within a few hours to days resulting in her left hemiparesis and necessitating hospital admission. The presence of right hemispheric meningeal enhancement, right hemispheric attenuation on EEG, right posterior quadrant discharges on interictal EEG, and bilateral posterior quadrant involvement with her seizures and improvement in hemiparesis following antiepileptic therapy help to support this hypothesis.

Levetiracetam and lacosamide were chosen for their ease of administration during status epilepticus and continued use on an outpatient basis. The patient in the present case does not have myoclonus, which is a known feature of SCA8, but levetiracetam was chosen to help with potential myoclonus as well [3]. Psychiatric symptoms have also been reported in patients with SCA8, but the present patient did not have any such complaints, which made the author feel more comfortable in choosing levetiracetam for her seizure therapy [3].

SCA8 is an uncommon type of SCA and can have a highly variable presentation (Table 1). Seizures are known to occur in the SCAs and must always be kept in mind as a possibility, especially due to the presence of a known neurodegenerative/genetic disease. Spells of altered awareness with or without automatisms are highly concerning for epileptic events, but nonepileptic causes such as movement disorders and psychogenic spells must always be kept in the differential diagnosis. Video EEG monitoring is essential for capturing these spells to confirm or exclude epileptic etiology and must be used accordingly. Antiepileptic therapy must be instituted when needed in such patients, and the choice of agent is determined by the ease of administration and comorbidities associated with them. Expert evaluation is highly recommended when treating such patients, and multidisciplinary collaboration for management involving epileptologists, movement disorder specialists, psychiatrists, physical medicine doctors, and others is highly advised. Genetic testing helps to confirm the diagnosis and establish care with appropriate specialists.

**Table 1:** Timeline of presentation

Age	Associated symptoms and presentation
10 years	Diagnosis of SCA8 with genetic testing following presentation with new-onset ataxia and gait difficulties
12 years	Spells of awareness and automatisms with confusion are first seen, not identified as epileptic at this time and not started on antiepileptic therapy
22 years – time of admission	Admitted with encephalopathy and left hemiparesis of unclear cause; routine EEG showing right occipital epileptiform discharges; started on low-dose levetiracetam
22 years – 2 days after admission	Observed to have confusional spells with automatisms; video EEG shows nonconvulsive status epilepticus of posterior quadrant onset; treated with higher doses of levetiracetam and lacosamide and status epilepticus resolves in 12 hours
22 years – 7 days after admission	Discharged to rehabilitation for 1 month with inpatient physical therapy for persistent left hemiparesis; return to mental baseline
22 years – 6 months after admission	Seen in clinic in follow-up; left hemiparesis has resolved and at mental baseline; seizure-free since discharge; remains on levetiracetam and lacosamide for seizure prophylaxis

*Abbreviations: EEG* Electroencephalography, *SCA8* Spinocerebellar ataxia 8

## Abbreviations

EEG: Electroencephalogram
SCA8: Spinocerebellar ataxia type 8
